# A retrospective serosurvey of selected pathogens in red foxes (*Vulpes vulpes*) in the Tuscany region, Italy

**DOI:** 10.1186/s13028-023-00699-6

**Published:** 2023-07-14

**Authors:** Gianmarco Ferrara, Giuseppina Brocherel, Beatrice Falorni, Roberta Gori, Ugo Pagnini, Serena Montagnaro

**Affiliations:** 1 Department of Veterinary Medicine and Animal Production, University of Naples, via Delpino 1, Federico, Naples, 80137 II Italy; 2 Istituto Zooprofilattico Sperimentale del Lazio e Della Toscana “M. Aleandri”, Arezzo, 52100 Italy

**Keywords:** Infectious diseases, Reservoirs, Surveillance, Vector-borne diseases, Wildlife, Zoonoses

## Abstract

The expansion of urbanization in natural environments increases interactions between wildlife, domestic animals, and humans. In Italy, the red fox (*Vulpes vulpes*) is one of the most common wild carnivores. This species can serve as a reservoir and sentinel host for several infectious diseases. We aimed to improve knowledge about the exposure of red foxes to selected zoonotic (*Anaplasma* spp, *Ehrlichia* spp., *Borrelia* spp., and hepatitis E virus) and carnivore-specific pathogens (canine parvovirus, canine distemper virus, pseudorabies virus, and *Dirofilaria* spp.) through a retrospective survey performed in the Tuscany region during the spring season of 2013. Using specific ELISAs and serum samples (n = 38) collected during a culling campaign, a prevalence of 2.6% for canine distemper virus, 18.4% for canine parvovirus, 5.2% for *Anaplasma* spp., 2.6% for *Ehrlichia* spp., 7.9% for *Dirofilaria* spp., 21.05% for hepatitis E virus, and 10.5% for pseudorabies virus was observed. Conversely, antibodies against *Borrelia* spp. were not identified in any of the animals. Our results revealed no significant sex-related differences in seroprevalence and confirmed hepatitis E virus as the most common pathogen in the analyzed samples. All of the animals that tested positive for tick-borne zoonotic agents presented ticks at the time of sampling. Our study confirms the exposure of red foxes in the Tuscany region to viral and bacterial infections raising medical and veterinary concern and indicating the need for large-scale surveillance to fully assess the epidemiological significance of these findings.

## Findings

Transmission of potentially zoonotic agents from wild to domestic animals and humans has increased in recent years due to a variety of factors, such as the growing number of wild animals, habitat fragmentation, invasion of the natural environment by wildlife, or occupation of natural environments by humans. This has contributed to ever increasing contact between human, domestic animals, and wildlife [1]. From a human health perspective, monitoring and surveillance of wildlife are critical to the containment of infections and represent a key aspect of conservation and management programs as well. Reports on the presence of infectious diseases (including zoonoses) in wild foxes are frequent. Different fox species are spread all over the world, including in Italy, where the red fox (*Vulpes vulpes*) is the main wild carnivore distributed throughout the country. Canine parvovirus (CPV), and canine distemper virus (CDV), are two viruses that foxes can transmit to dogs (and vice versa) [2]. CPV is responsible for acute gastrointestinal forms (mainly haemorrhagic), which can be fatal, especially when affecting puppies, in both domestic and wild carnivores. CDV causes similar mortality rates to CPV, although it displays slightly different tissue tropism, replicating in the respiratory and nervous systems in addition to the gastrointestinal tract. Due to the high mortality rates, these viral infections should also be considered in wildlife monitoring, as they may pose a potential threat to biodiversity conservation, and a risk for the re-emergence of infections in the domestic dog population [3]. Another concerning viral infection to which wild animals, especially carnivores, are predisposed is pseudorabies (PRV), a disease that is commonly found in swine and in dead-end hosts (other mammals) [4]. In swine the disease is characterized by respiratory symptoms and reproductive disorders, while in carnivores it is characterized by intense itching and encephalomyelitis, which can be fatal [5].

The red fox is also susceptible to several zoonoses. Particularly, canine vector-borne diseases (CVBD), including *Anaplasma* spp., *Ehrlichia* spp., and *Borrelia* spp., are health issues for both humans and animals. As a worldwide spread disease, CBVD has become an increasing concern, especially due to global warming and anthropogenic factors [1]. These infections are transmitted by ixodid ticks (*Ixodes* spp. and *Rhipicephalus* spp.) and sometimes evolve into a severe disease in domestic dogs. However, these diseases usually result in subclinical infections in wild carnivores [1]. *Dirofilaria immitis* is a nematode responsible for another arthropod-borne disease. In the adult stage, this parasite lives in the pulmonary arteries and heart of infested canids and is transmitted by mosquitoes. In the past, this disease was confined to Po Valley in Northern Italy, however, nowadays it has become endemic throughout Italy. The disease is characterized by cough, anemia, and, in the severest cases, heart failure. The fox is also described in the epidemiological cycle of hepatitis E virus, and there are numerous reports of its occurrence in this species [6]. Considering the wide distribution of this species in Italy and the increasing overlap of habitats between urban and wild populations, the purpose of this study is to provide information on the past exposure of the aforementioned infections to red foxes in the Tuscany region, central Italy.

A total of 38 red foxes were sampled in different sampling sites in Tuscany region, during the red fox culling campaign conducted in the provinces of Arezzo and Pistoia in the spring of 2013 (“Procedura di controllo della fauna selvatica in regione Toscana”, art.37 della L.R. 3/1994). Blood was collected by intracardiac puncture, then centrifuged at 1500 g for 15 min, and finally stored at -20 °C until being analyzed. We employed a panel of different ELISAs: (i) SNAP ® 4Dx® plus (IDEXX, [US]) was used for the detection of antibodies against *Anaplasma phagocytophilum* and *A*. *platys*, *Borrelia burgdorferi*, *Ehrlichia canis* and *E. ewingii* and the antigen of *Dirofilaria immitis*; (ii) the indirect distemper IgG Ab ELISA and parvo IgG Ab ELISA (Agrolabo, [Italy]) tests were used to detect the IgG against CDV and CPV, respectively; (iii) the combination of both competitive PRV/ADV gE Ab and PRV/ADV gB Ab (IDEXX, [US]) ELISAs was used to detect antibodies against PRV and to evaluate the potential exposure of the animals to vaccine strains [4]; (iv) and the multispecies and double-antigen ELISA hepatitis E virus (HEV) Ab Version ULTRA (Dia.Pro, [Italy]) was used for qualitative determination of total antibodies against HEV. The manufacturer’s instructions were followed for all the tests employed, and in the case of the ELISAs, the samples were determined as positive or negative based on the cut-off indicated in the assay’s instructions. These tests, although validated for other species, have been successfully used in wild carnivores in other epidemiological studies [7, 8]. The seroprevalence and corresponding 95% confidence intervals (CIs) were calculated by using the binomial exact test. Comparisons in seroprevalence according to sex were assessed by applying the Fisher’s exact test. All the statistical analyses were conducted with MedCalc Statistical software (version 16.4.3) and statistical significance was considered when the P-value was < 0.05.

We examined 38 animals, of which 16 were males (42.1%) and 22 females (57.9%), and 26.3% of the overall animals were parasitized by ticks at the time of sampling. Twenty out of 38 individuals tested positive for at least one pathogen, while 18 of the animals yielded negative results. The highest seroprevalence levels were registered for most of the viral agents assessed, mainly HEV, CPV, and PRV (Table 1). Moreover, six out of 38 individuals yielded positive results for vector-borne pathogens, with *Anaplasma* spp. and *Dirofilaria* spp., being the agents to which the individuals were most often exposed. By contrast, no animal tested positive for antibodies against *Borrelia* spp. (Table 1). We also observed different patterns of pathogen co-exposure in four animals: CVD + PRV + *Dirofilaria* spp. (n = 1), PRV + *Anaplasma* spp. (n = 1), HEV + PRV (n = 1) and CPV + PRV (n = 1). None of the pathogen exposures displayed a predisposition related to animal sex (Fisher exact test, P > 0.05; Table 2). All three tick-borne disease positive animals presented ticks on their bodies at the time of sampling.

**Table 1:** Number and prevalence of selected pathogens in red foxes (n = 38) in Tuscany region during the spring of 2013

Agent	Number of positive cases and %	95%CI
Canine parvovirus	7 (18.4)	6.1–30.7
Canine distemper virus	1 (2.6)	0–7.7
*Ehrlichia sp.*	1 (2.6)	0–7.7
*Anaplasma sp.*	2 (5.6)	0–12.4
*Borrelia sp.*	0 (0)	0
*Dirofilaria immitis*	3 (7.9)	0–16.5
Pseudorabies virus	4 (10.5)	0.8–20.3
Hepatitis E virus	8 (21.05)	8.1–34

**Table 2:** Seroprevalence for selected pathogens in red foxes according to sex in Tuscany region

Agent	Number of seropositive males and %	Number of seropositive females and %	χ^2^	*P*
Canine parvovirus	3 (18.7)	4 (18.2)	0.002	0.96
Canine distemper virus	0 (0)	1 (4.5)	0.75	0.39
*Ehrlichia sp.*	0 (0)	1 (4.5)	0.75	0.39
*Anaplasma sp.*	1 (6.2)	1 (4.5)	0.05	0.81
*Dirofilaria immitis*	0 (0)	3 (13.6)	2.37	0.12
Pseudorabies virus	1 (6.2)	3 (13.6)	0.53	0.46
Hepatitis E virus	3 (18.7)	5 (22.7)	0.09	0.77

Our findings confirmed the exposure of wild carnivores to a variety of infections, as described in other studies conducted in other countries. For example, the circulation of CPV and CDV in wild carnivores has been documented in several continents, including Southern America (namely Argentina, and Chile), Central America (the Western United States and south-eastern Colorado), and Europe (Spain, Luxembourg, Germany, Portugal, and Scandinavian countries) [9–17]. In all these studies, the prevalence of CDV (range 3–36%) was always lower than that of CPV (range 30–69%). This variation can be explained by the different lethality of CPV and CDV in domestic and wild species. In fact, whereas a lower mortality has been described for CPV in wildlife, the same cannot be stated for CDV, which displays the same lethality regardless of host. Already in 2006, outbreaks of distemper were reported in the red fox population in northern and north-eastern Italy [18]. The outbreak reoccurrence was also observed in 2013–2015 (coinciding with our sampling time), when 32% of 548 wild carnivores were found positive for direct immunofluorescence, and recently in 2018–2020 [19, 20]. CPV has also been described as the leading cause of death in coyotes, wolves, and foxes in Yellowstone Park (US) [21].

Previous studies have identified red foxes as potential maintenance hosts for *Ehrlichia* spp. and *Anaplasma* spp [1, 23]. However, we observed a low seroprevalence for both pathogens, which may be explained by the sensitivity of the analytical techniques employed and by the time period required for the hosts to generate a detectable immunological response. The effect of tick-borne infections on the health and conservation of wild carnivores is not widely recognized. Ehrlichiosis and anaplasmosis have been documented in wild carnivores all over the world, although there have been few clinical experiences in wild animals [22, 23]. The absence of Borrelia-positive animals could be explained by the low sample size examined and because of the role of red foxes within the epidemiological cycle of *Borrelia* spp., that has been discussed in the last few years. It has been demonstrated that the reduction of some predators which restrict the quantity of essential rodent reservoirs may have an indirect effect on the occurrence of Lyme borreliosis in humans. In fact, a decline in the fox population could possibly lead to an increase in Lyme disease, as it has also been demonstrated for other tick-borne infections with similar trends [23, 24]. A study in Ontario using the same rapid kit found that 1.5% of wild canids tested positive for *B. burgdorferi* antibodies and none for *Anaplasma* spp. or *Ehrlichia* spp [8]. Higher prevalences have been described among stray dogs in Italy (16% for *Ehrlichia* spp. and 7.8% for *Anaplasma* spp.) [25].

Wild carnivores represent susceptible hosts for *Dirofilaria* spp. due to the lack of protection by chemoprophylaxis. Similar prevalence values were observed in other European countries, such as Hungary (3.7%) and Serbia (1.55%), while a study performed in California reported a prevalence of up to 100% [26–28].

The spread of antibodies against PRV reflected the epidemiological situation of the country in other species (the disease has been routinely described in swine and canids) [4, 5].

The most prevalent infection was HEV. Antibodies against this virus have been reported in several species of domestic and wild carnivores, but the excretion of viral RNA in feces has been demonstrated only in wildlife, emphasizing the need to monitor this infection to provide new information about its epidemiology [6, 29, 30]. Other studies focusing on the red fox population uncovered an annual oscillation in seroprevalence between 40 and 100% in Germany [6]. The observed differences are not only due to different epidemiological situations, but also to intrinsic factors such as the type of test used, the sampling period, and the size of the analyzed sample.

This study confirmed the exposure of the red fox population to primary zoonotic agents and raised questions about the impact of increasing contact between humans and domestic animal populations with wildlife. On the other hand, this study highlights the potential influence of infectious diseases in carnivore conservation. In fact, due to their high lethality, CDV and CPV are identified as potential threats that can hinder wildlife conservation and cause changes in population dynamics.

Because this study used a small sample size and outdated samples for the investigated geographic region, larger-scale investigations are needed to assess the current epidemiological situation. Continuous wildlife monitoring is required to improve our understanding of the human/animal/environment axis with the intent of keeping full compliance with the concept of One Health.

## Data Availability

The datasets used during the current study are available from the corresponding author on reasonable request.

## Declarations

### Prior publication

Data have not been published previously.
